# Supplementary material for: Predicting CoVID-19 community mortality risk using machine learning and development of an online prognostic tool
Source: PeerJ. 2020 Sep 28;8:e10083. doi: 10.7717/peerj.10083 (PMC7528809; doi:10.7717/peerj.10083)
Supplement: Supplemental Information 1 [file peerj-08-10083-s001.docx]

**Supplemental Table S1. Hyperparameter tuning of machine learning algorithms using grid search**

| **Algorithm** | **Hyperparameters tested (range)** | **Best fit hyperparameters** | |
| --- | --- | --- | --- |
|  |  | **SMOTE^#^** | **ADASYN^*^** |
| **Logistic regression** | Regularization (l1, l2) | l2 | l2 |
|  | Error rate (0.001, 0.009, 0.01, 0.09, 1, 5, 10, 25) | 25 | 25 |
| **Support vector machine** | Kernel (linear, radial, polynomial, sigmoid) | Radial | Radial |
|  | Gamma (0.0001, 0.0005, 0.001, 0.005, 0.01, 0.1) | 0.1 | 0.1 |
|  | Error rate (0.001, 0.01, 0.1, 1, 10, 100) | 1 | 100 |
| **K nearest neighbor** | Number neighbors (1-30) | 29 | 27 |
|  | Leaf size (1-50) | 1 | 39 |
|  | Weights (uniform, distance) | Distance | Distance |
|  | Metric (Euclidean, Manhattan) | Manhattan | Manhattan |
| **Random forest** | Criterion (Gini, entropy) | Gini | Gini |
|  | Estimators (20-81) | 50 | 60 |
|  | Minimum leaf samples (1-3) | 1 | 1 |
|  | Minimum samples to split (3-7) | 6 | 3 |
|  | Maximum depth (5, 8, 15, 25, 30) | 25 | 25 |
| **Gradient boosting** | Learning rate (0.15,0.1,0.05,0.01,0.005,0.001) | 0.15 | 0.15 |
|  | Estimators (20-100) | 80 | 80 |
|  | Minimum leaf samples (1-3) | 1 | 1 |
|  | Minimum samples to split (3-7) | 3 | 4 |
|  | Maximum depth (3, 5, 8) | 8 | 8 |
|  | Maximum features (2-7) | 2 | 5 |

# SMOTE – Synthetic minor oversampling technique; * ADASYN – Adaptive synthetic sampling
